# Ethanolic Extract of Acanthopanax koreanum Nakai Alleviates Alcoholic Liver Damage Combined with a High-Fat Diet in C57BL/6J Mice

**DOI:** 10.3390/molecules21060681

**Published:** 2016-05-24

**Authors:** Haein Kim, Minyoung Park, Jae-Ho Shin, Oran Kwon

**Affiliations:** 1 Department of Nutritional Science and Food Management, Ewha Womans University, 52 Ewhayeodae-gil, Seodaemun-gu, Seoul 03760, Korea; khi211@ewhainan.net (H.K.); hanabi_223@daum.net (M.P.); 2 Department of Biomedical Laboratory Science, Eulji University, Seongnam, Gyeonggi-do 13135, Korea; shinjh@eulji.ac.kr

**Keywords:** *Acanthopanax koreanum* Nakai, interactive effect of alcohol and high-fat diet, hepatic damage

## Abstract

Alcoholic and nonalcoholic liver steatosis have an indistinguishable spectrum of histological features and liver enzyme elevations. In this study, we investigated the potential of the ethanolic extract of *Acanthopanax koreanum* Nakai (AK) to protect against experimental alcoholic liver disease in a mouse model that couples diet and daily ethanol bolus gavage. Fifty-six C57BL/6J mice were randomly divided into seven groups: normal control (NC), alcohol control (AC), alcohol/HFD control (AH), low-dose (1%) AK in alcohol group (ACL), high-dose (3%) AK in alcohol group (ACH), low-dose AK in alcohol/HFD group (AHL), and high-dose AK in alcohol/HFD group (AHH). The AH group showed more severe damage than the AC group in terms of biochemical and molecular data that were observed in this study. The administration of AK exerted remarkable effects in: plasma ALT (*p* < 0.0001), total lipid (*p* = 0.014), TG (*p* = 0.0037) levels; CPT-1α (*p* = 0.0197), TLR4 (*p* < 0.0001), CD14 (*p* = 0.0002), IL-6 (*p* = 0.0264) and MCP-1 (*p* = 0.0045) gene expressions; and ALDH (*p* < 0.0001) and CAT (*p* = 0.0076) activities. The data suggested that at least the high dose AK might confer protection against alcoholic liver damage combined with an HFD by accelerating lipid oxidation and alcohol metabolism and by suppressing the inflammatory response, including the TLR pathway.

## 1. Introduction

Chronic and excessive alcohol consumption can lead to the development of alcoholic liver disease (ALD), which remains one of the most silent pathophysiological conditions worldwide [1,2]. ALD includes a broad spectrum of disorders, ranging from simple steatosis to severe steatohepatitis, cirrhosis and hepatocellular diseases [3]. A high-fat diet (HFD) in the absence of substantial alcohol consumption has also been found to produce dyslipidemic syndrome referred to as nonalcoholic fatty liver disease (NAFLD). The NAFLD also encompasses mild hepatic steatosis to steatohepatitis [4]. In modern societies, people frequently drink alcohol with high-calorie foods, which may aggravate liver damage [5]. Demori *et al*. [4] suggested that acute alcohol administration to rats combined with HFD produced oxidative stress in a liver already affected by lipid accumulation. Gäbele *et al.* [6] demonstrated that alcohol with an HFD acted synergistically on hepatic injury via enhanced TLR4 signaling in a Balb/c mice model.

*Acanthopanax koreanum* Nakai is a plant indigenous to Jeju Island, Korea [7]. Traditionally, it has been used as a tonic and sedative agent [8]. Recent investigations of the chemical composition of *Acanthopanax koreanum* Nakai revealed that lupane triterpene and their glycosides, diterpenes (acanthoic acid and kaurenoic acid) and lignans (eleutheroside **B** and **E**) are major metabolites [9,10]. Several lines of evidence have been accumulated, suggesting that each active ingredient of *Acanthopanax koreanum* Nakai may have health benefits, such as anti-inflammatory [11], cytotoxic [12], hepatoprotective [8], immunomodulatory [13] and antioxidant [14] effects *in vivo* and *in vitro*. For most studies, including our own, the hepatoprotective effect of *Acanthopanax koreanum* Nakai has been proven by using a chemical (acetaminophen, tetrachloride or d-galactosamine/lipopolysaccharide)-induced hepatic toxicity animal model [7,15,16,17]. At present, however, no studies have been conducted to investigate its hepatoprotective role, especially in an alcohol-intoxicated animal model that couples diet and daily ethanol bolus gavage.

Therefore, in the present study, we explored the hypothesis that *Acanthopanax koreanum* Nakai may have a protective action against the combination of ALD and NAFLD. To test this hypothesis, different doses of ethanolic extract of *Acanthopanax koreanum* Nakai (AK) were administered in a mice model that couples an HFD and daily ethanol bolus gavage. Then, the protective effects and mode of action of AK were investigated by evaluating its potential impact on lipid and alcohol metabolism, as well as anti-inflammatory properties, including TLR4 signaling.

## 2. Results

### 2.1. Effect of AK on Alcohol and HFD-Induced Hepatic Steatosis

During the experimental period, food intakes were significantly decreased in the alcohol-fed groups and more significantly decreased in the groups treated with alcohol combined with an HFD, when compared to the normal control (NC) group (*p* < 0.0001). AK treatment did not restore the reduced food intake. However, the final body weights were not significantly different among groups, reflecting the fact that daily calorie intake was similar in all diet groups (Table 1).

**Figure 1:**
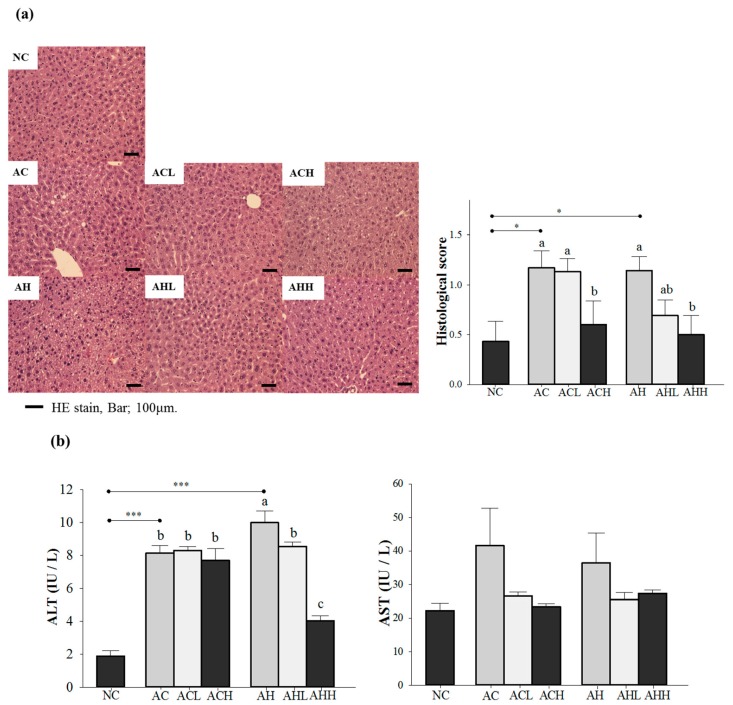
Effects of AK on hepatic damage in C57BL/6J mice fed with alcohol and HFD: (**a**) histopathological findings (hematoxylin-eosin staining) and (**b**) serum ALT and AST levels. Each value is expressed as the mean ± S.E. NC, normal control; AC, alcohol control; ACL, low-dose (1%) AK in alcohol group; ACH, high dose (3%) AK in alcohol group; AHC, alcohol/HFD control; AHL, low-dose AK in alcohol/HFD group; and AHH, high-dose AK in alcohol/HFD group. AK, ethanolic extract of *Acanthopanax koreanum* Nakai; HFD, high-fat diet; ALT, alanine aminotransferase; AST, aspartate aminotransferase. Mean bars with different alphabets are significantly different among alcohol ingestion groups at *p* < 0.05 with Duncan’s multiple comparison tests. * *p* < 0.05 and *** *p* < 0.0001 (AC and AH to NC).

We confirmed the hepatic steatosis and damage by hematoxylin-eosin (H & E) staining and an increase of plasma alanine aminotransferase (ALT) level in either the AC or AH group, while normal lobular architecture and cell structure were observed in the NC group (*p* = 0.0041 for histological score; and *p* < 0.0001 for ALT). Changes of plasma aspartate aminotransferase (AST) level did not reach statistical significance. A high-dose AK administration significantly reduced the histological to near normal level groups administered either alcohol alone or alcohol with an HFD (*p* = 0.0156) (Figure 1a). However, the inhibitory effect of AK against the increase of ALT was more pronounced in groups treated with alcohol combined with an HFD than in groups treated with alcohol only, showing a dose-dependent suppression (*p* < 0.0001) (Figure 1b).

### 2.2. Effect of AK on Lipid and Alcohol Metabolism in Alcohol and HFD-Induced Hepatic Steatosis

**Figure 2:**
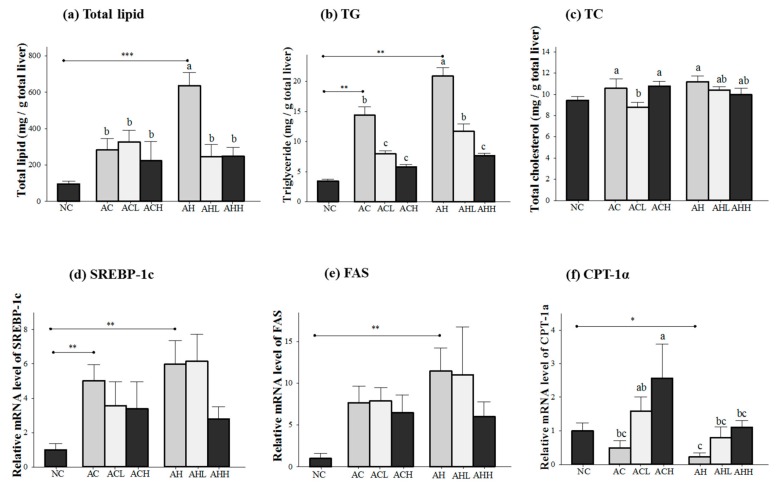
Effects of AK on hepatic lipid metabolism in C57BL/6J mice fed with alcohol and HFD: (**a**) total lipid; (**b**) TG; (**c**) TC; (**d**) SREBP-1c; (**e**) FAS, and (**f**) CPT-1α. Each value is expressed as the mean ± S.E. NC, normal control; AC, alcohol control; ACL, low-dose (1%) AK in alcohol group; ACH, high dose (3%) AK in alcohol group; AHC, alcohol/HFD control; AHL, low-dose AK in alcohol/HFD group; and AHH, high-dose AK in alcohol/HFD group. AK, ethanolic extract of *Acanthopanax koreanum* Nakai; HFD, high fat diet; TG, triacylglycerol, TC, total cholesterol; SREBP-1c, sterol regulatory element-binding protein-1c; FAS, fatty acid synthase; CPT-1α, carnitine palmitoyltransferase 1α. Mean bars with different alphabets are significantly different among alcohol ingestion groups at *p* < 0.05 with Duncan’s multiple comparison tests. * *p* < 0.05, ** *p* < 0.01 and *** *p* < 0.001 (AC and AH to NC).

Figure 2 shows the changes of lipid concentration and gene expressions in the liver. Total lipid and triacylglycerol (TG) levels in the liver were increased in both AC and AH groups compared to the NC group, but a more significant increase was found in the AH group (*p* < 0.0001 for total lipid; and *p* = 0.0003 for TG). AK administration was effective at attenuating the increase in total lipid (*p* = 0.0154) and TG (*p* < 0.0001) in a dose-dependent manner. Two-way ANOVA analysis revealed that there was a significant interaction between AK and HFD for total lipid (*p* = 0.014) and TG (*p* = 0.0037) (Figure 2a,b). In contrast, the hepatic TC level was not affected in the AC group, but significantly increased in the AH group only compared to the NC group (*p* = 0.0191) to a much lesser degree. The effect of AK on hepatic TC level was also minimal (*p* = 0.0475) and did not show either dose-dependent behavior or an interaction (Figure 2c).

Genes for lipogenesis (sterol regulatory element-binding protein-1c, SREBP-1; and fatty acid synthase, FAS) were significantly overexpressed in the AC and AH groups compared to the NC group (*p* = 0.0048 for SREBP-1c; and *p* = 0.0053 for FAS). Although a decrease was found in the ACH and AHH groups, it did not reach statistical significance (Figure 2d,e). Carnitine palmitoyltransferase 1α (CPT-1α) gene expression was decreased in both AC and AH groups, but only the AH group showed a statistical significance compared to the NC group (*p* = 0.03). AK administration restored CPT-1α gene expression in a dose-dependent manner (*p* = 0.0197). Two-way ANOVA analysis revealed a significant impact of AK (*p* = 0.0101) and HFD (*p* = 0.0197), but no interaction between AK and HFD (Figure 2f).

**Figure 3:**
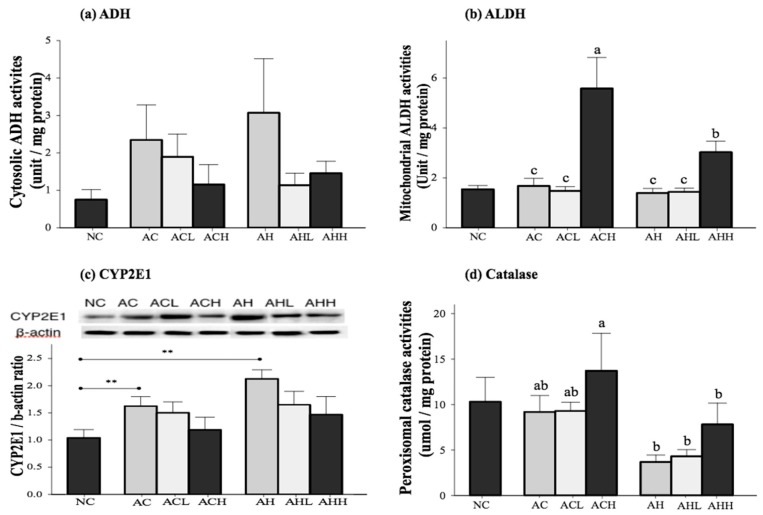
Effects of AK on alcohol metabolism in C57BL/6J mice fed with alcohol and HFD: (**a**) ADH; (**b**) ALDH; (**c**) CYP2E1; and (**d**) Catalase. Each value is expressed as the mean ± S.E. NC, normal control; AC, alcohol control; ACL, low-dose (1%) AK in alcohol group; ACH, high dose (3%) AK in alcohol group; AHC, alcohol/HFD control; AHL, low-dose AK in alcohol/HFD group; and AHH, high-dose AK in alcohol/HFD group. AK, ethanolic extract of *Acanthopanax koreanum* Nakai; HFD, high fat diet; ADH, alcohol dehydrogenase; ALDH, acetaldehyde dehydrogenase; CYP2E1, cytochrome P450 2E1. Mean bars with different alphabets are significantly different among alcohol ingestion groups at *p* < 0.05 with Duncan’s multiple comparison tests. ** *p* < 0.01 (AC and AH to NC).

The activities of the primary enzymes involved in alcohol metabolism (alcohol dehydrogenase, ADH; acetaldehyde dehydrogenase, ALDH; cytochrome P450 2E1, CYP2E1; and catalase, CAT) were measured in the liver (Figure 3). Cytosolic ADH activity was increased in the AC and AH groups and decreased by AK administration, although the changes did not reach statistical significance (Figure 3a). Mitochondrial ALDH activity was not different among the NC, AC and AH groups, but was significantly increased by a high-dose administration of AK in the ACH and AHH groups (*p* < 0.0001) (Figure 3b). Microsomal CYP2E1 expression was significantly increased in the AC and AH groups compared to the NC group (*p* = 0.0007). Although AK administration suppressed microsomal CYP 2E1 expression, there was no statistical significance among the groups (Figure 3c). Peroxisomal CAT activity was not significantly changed in the AC group, but showed a slight decrease in the AH group compared to the NC group (*p* = 0.0546). The CAT activity was increased with administration of AK at a high dose, showing the highest activity in the ACH group (*p* = 0.0076). Two-way ANOVA analysis revealed a significant impact from HFD (*p* = 0.0008) and a moderate impact from AK (*p* = 0.0801), but no interaction between AK and HFD (Figure 3d).

### 2.3. Effect of AK on Hepatic Inflammatory Responses in Alcohol and HFD-Induced Hepatic Steatosis

**Figure 4:**
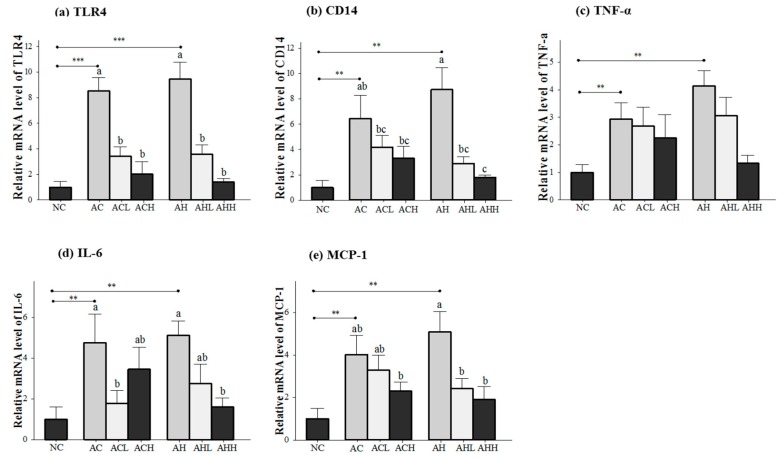
Effects of AK on inflammatory response in the liver of C57BL/6J mice fed with alcohol and HFD: (**a**) TLR4; (**b**) CD14; (**c**) TNF-α; (**d**) IL-6; and (**e**) MCP-1. Each value is expressed as the mean ± S.E. NC, normal control; AC, alcohol control; ACL, low-dose (1%) AK in alcohol group; ACH, high dose (3%) AK in alcohol group; AHC, alcohol/HFD control; AHL, low-dose AK in alcohol/HFD group; and AHH, high-dose AK in alcohol/HFD group. AK, ethanolic extract of *Acanthopanax koreanum* Nakai; HFD, high fat diet; TLR, toll-like receptor; CD14, cluster of differentiation 14; TNF-α, tumor necrosis factor-α; IL-6, interleukin-6; MCP-1, monocyte chemotactic protein-1. Mean bars with different alphabets are significantly different among alcohol ingestion groups at *p* < 0.05 with Duncan’s multiple comparison tests. ** *p* < 0.01 and *** *p* < 0.001 (AC and AH to NC).

Genes related to the inflammatory response (toll-like receptor 4, TLR4; cluster of differentiation 14, CD14; tumor necrosis factor-α, TNFα; interleukin-6, IL-6; and monocyte chemotactic protein-1, MCP-1) were measured in the liver (Figure 4a,e), because the consequences of alcohol and HFD include the generation of harmful compounds in the liver. Gene expression levels in the AC and AH groups were all significantly increased relative to the NC groups (*p* < 0.05), with a bigger change in the AH group than in the AC group. AK administration showed a dose-dependent suppression in general, but statistical significance was found only for the TLR4 (*p* < 0.0001), CD14 (*p* = 0.0002), IL-6 (*p* = 0.0264) and MCP-1 (*p* = 0.0045) genes. Moreover, two-way ANOVA analysis revealed a significant impact from AK on CD14 (*p* = 0.002), IL-6 (*p* = 0.006) and MCP-1 (*p* = 0.0045).

## 3. Discussion

The goal of this study was to test the hypothesis that AK administration may rescue liver damages induced by alcohol and HFD intake in male C57BL/6J mice. We confirmed the previous finding that chronic consumption of alcohol with an HFD produces an appropriate model for excessive nutritional and alcohol abuse [18]. Alcohol administration for four weeks caused significant hepatic damage as evidenced by increases in hepatic lipid droplets, plasma ALT level and inflammatory cytokine level, which were aggravated with the combination of HFD. Beneficial changes were seen in almost all observed markers in the groups that received AK compared to the control groups, thus supporting the hypothesis for the first time.

The leakage of cellular enzymes (AST and ALT) is a prominent sign of hepatic injury [19]. We found that plasma ALT level was more specific for liver damage than plasma AST level and also appeared to be compatible with the histologic findings in the liver. This result is consistent with the previously published data in that alcoholic hepatitis altered the plasma ALT level more than the plasma AST level after several days of exposure to alcohol [20]. AK administration was sufficient to protect against changes in hepatic fatty liver and plasma ALT levels in mice receiving both alcohol and HFD. In contrast, significant changes were not achieved in mice treated with alcohol administration only. The observed differences in AK effects are likely related to the additive effects of AK on lipid and alcohol metabolism. 

Consistent with the previous report of Purohit *et al.* [21], we confirmed that alcohol and HFD consumption significantly increased SREBP-1 and FAS gene expressions and decreased CPT-1α gene expression. While AK administration did not suppress lipogenesis gene expression, it did cause a significant increase in lipolysis gene expression. This result indicates that an excessive TG accumulation by alcohol overload and HFD can be successfully reduced in the liver in the presence of AK via stimulation through β-oxidation.

The major pathway for alcohol metabolism in the liver involves its conversion to aldehyde by cytosolic ADH and rapid oxidation of the aldehyde to acetic acid by mitochondrial ALDH. No significant changes in the ADH activity were found either in the alcohol and HFD fed groups or in the AK administration groups. This is supported by the fact that ADH is not inducible as a result of either alcohol consumption or other dietary conditions [22]. However, interestingly, AK administration significantly increased ALDH activities at a high dose, implying that AK accelerated alcohol metabolism. Two additional pathways of acetaldehyde generation involve microsomal ethanol oxidizing systems, predominantly via CYP2E1, and peroxisomal CAT [23]. CYP2E1 is inducible by alcohol and plays an important role in eliminating alcohol at high blood alcohol levels [24]. CYP2E1 is also induced by HFD [25]. However, it is important to note that oxygen activation by CYP2E1 produces reactive oxygen species (ROS) and, thus, causes mitochondrial dysfunction and endoplasmic reticulum stress in the liver [26]. CAT is also inducible and oxidizes alcohol to aldehyde in a hydrogen peroxide-dependent fashion. A significant induction of CYP2E1 gene expression was found in the alcohol- and HFD-fed groups, and some favorable reversals were observed in the AK groups, although the changes did not reach statistical significance. In contrast, there was a slight decrease in CAT activity in the alcohol group, reflecting the minor role of CAT in alcohol metabolism [27]. The decrease was more evident in the alcohol- and HFD-fed groups. However, AK administration was sufficient to restore CAT activity to the normal control level. 

The gut-liver axis seems to play an important role in the induction of steatosis and inflammation in the liver [28]. Alcohol and HFD contribute to increased gut permeability, potentially amplifying the rise of portal endotoxin levels [29]. At the cellular level, gut-derived endotoxin is recognized by various receptors, including CD14. However, CD14 lacks a transmembrane domain, and therefore, TLR4, a second receptor, is required to activate Kupffer cells, leading to the induction of chemokines (MCP-1) and upregulation of the inflammatory cascade [28]. Alcohol and HFD consumption induced significant increases in TLR4, CD14 and pro-inflammatory cytokines, as expected. The potential for a less inflammatory effect was found in the AK administration groups. This improvement was greater in groups treated simultaneously with alcohol and HFD than in groups treated with alcohol only.

## 4. Materials and Methods 

### 4.1. AK Preparation

The AK was provided by Jeju Technopark (Jeju, Korea). Briefly, the stem and root (8:2) of *Acanthopanax koreanum* Nakai were ground and dried in the shade, followed by extraction with 70% ethanol at 60 °C for 15 h. Extracts were filtrated, concentrated and freeze-dried to make a dried powder for use in this study. The AK was standardized with acanthoic acid (AA) at 1.95% using high-performance liquid chromatography (Waters Alliance HPLC systems, Waters corporation, Milford, MA, USA) with a photodiode array detector and a Cadenza C_18_ column (250 mm × 4.6 mm, 3 µm) at 30 °C. The mobile phase consisted of acetonitrile in 0.5% acetic acid solution (10:90, *v*/*v*, Solvent A) and acetonutrile (Solvent B) for the isocratic elution (A:B = 20:80) over 20 min at a flow rate of 1 mL/min.

### 4.2. Animals and Diets

Eight-week-old male C57BL/6J mice were obtained from Jung-Ang Lab Animal (Seoul, Korea). Animals were housed individually at a temperature of 23 ± 1 °C with 12/12 h light/dark cycles and 45% ± 5% humidity and were maintained with free access to water and standard food for 7-day adaptation. Then, the animals were randomly divided into 7 groups (*n* = 8 per group) and fed the following diets for 4 weeks: normal control (NC), alcohol control (AC), low-dose (1%) AK in alcohol group (ACL), high-dose (3%) AK in alcohol group (ACH), alcohol/HFD control (AH), low-dose AK in alcohol/HFD group (AHL), and high-dose AK in alcohol/HFD group (AHH). Ethanol (50%, *v*/*v*) was provided by oral gavage every day at the same time of the day for 4 weeks with a gradual increase in the dosage (2.5–4 g/kg body weight) for sequential stimulation. The NC and AC groups were fed a commercial mouse chow (10% energy from fat) (Teklad Diet TD6416, Madison, WI, USA), whereas the AH groups were fed a high-fat commercial mouse chow (45% energy from fat) (Harlan Teklad Diet TD6415). The AK was mixed in a high-fat diet at 1% or 3% (*w*/*w*) and fed throughout the experiment.

At the end of the experiment, animals were fasted overnight, euthanized by CO_2_ inhalation, and exsanguinated by cardiac puncture. The blood sample was centrifuged (3000× *g*, 4 °C, 30 min) to separate the plasma and then stored at −80 °C before analysis. To examine the histological changes, parts of the livers were preserved with phosphate-buffered formalin. The remaining samples were snap frozen in liquid nitrogen and stored at −80 °C until analysis. The experimental protocol was approved by the Institutional Animal Care and Use Committee (IACUC) of Ewha Womans University (Seoul, Korea), and all experimental procedures were conducted in compliance with the guidelines of Ewha Womans University for the care and use of laboratory animals. 

### 4.3. Histological Analysis

The formalin-fixed liver tissue was dehydrated in a 70%–100% gradient of ethyl alcohol, dealcoholized in xylene and embedded in paraffin for sectioning. The 5-µm sections were deparaffinized in xylene, rehydrated in a reverse-gradient series of ethyl alcohol and stained with H & E. Liver steatosis was graded by a pathologist to detect the presence of fat, necrosis, fibrosis and inflammation using the standards proposed by Dixon for assessing changes in fat and inflammation [30]. The score is defined as follows: 0 represents no ballooning; 1 represents mild steatosis (<33%) and occasional ballooning; 2 represents moderate steatosis (33%–67%) and obvious ballooning; and 3 represents severe steatosis (>67%) and marked ballooning.

### 4.4. Preparation of Cytosol, Microsome, Peroxisome and Mitochondria Fractions in the Liver

Liver tissue was homogenized with 10 volumes of 1.15% potassium chloride buffer containing 10 mM phosphate and 1 mM EDTA (pH 7.4) and fractionated by method of Volk *et al.* [31]. The homogenate was first centrifuged at 1900 rpm for 10 min at 4 °C. Then, the supernatant from this was centrifuged at 10,000 rpm for 20 min at 4 °C to precipitate the mitochondrial fraction. The resulting supernatant was then centrifuged at 17,000 rpm for 20 min at 4 °C to precipitate the peroxisome fraction. Then, the supernatant was centrifuged at 34,000 rpm for 1 h at 4 °C to pellet the microsomal fraction. The supernatant (cytosol) was used for ADH activities; the mitochondria fraction was used for ALDH; the microsome fraction was used for CYP2E1; and the peroxisome fraction was used for CAT.

### 4.5. Biochemical Assays

Plasma AST and ALT activities were measured using a commercial kit (Asan Pharmaceutical, Seoul, Korea). For the determination of lipid metabolism in the liver, total lipid was extracted by Folch’s method [28]. Triacylglycerol and cholesterol levels were determined using an enzymatic assay kit (Asan Pharmaceutical). Cytosolic ADH activity was determined by Bonnichsen’s method [32]; mitochondrial ALDH activity was determined by Koivusalo’s method [33]; and peroxisomal CAT activity was determined by Johansson’s method [34].

### 4.6. Western Blot Analysis

The microsome fraction (equivalent to 30 μg of protein) was separated utilizing 10% sodium dodecyl sulfate polyacrylamide gel electrophoresis, transferred to polyvinylidene difluoride membranes (Bio-Rad, Hercules, CA, USA) and blocked with 5% skim milk in Tris-buffered saline containing 0.1% Tween 20 (TBS-T) at room temperature for 1 h. The membrane was washed three times in TBS. Subsequently, the membrane was probed overnight with mouse anti-CYP2E1 (1:1000, Abcam, Cambridge, MA, USA) and mouse anti-β-actin (1:500, Santa Cruz Biotechnology, Santa Cruz, CA, USA) in 5% bovine serum albumin in TBS-T at 4 °C. The immunoreactive antigen was then recognized using anti-rabbit or anti-mouse horseradish peroxidase-labeled secondary antibodies (1:2000, Santa Cruz Biotechnology, Inc., Dallas, TX, USA) in 5% BSA in TBS-T for 2 h at room temperature. The blotted membrane was visualized with the West One Western Blot Detection System (iNtRON Biotechnology, Seongnam, Korea) and detected with the ChemiDoc XRS system with Image Lab software (Bio-Rad). To ensure equal loading, the relative target protein levels were normalized relative to β-actin.

### 4.7. Total RNA Isolation and Quantitative Real-Time Reverse Transcription Polymerase Chain Reaction Analysis

Total RNA was extracted from the liver using TRIzol (Invitrogen, Carlsbad, CA, USA). The RNA concentration and quality were determined with a BioSpec-nano spectrophotometer (Shimadzu, Kyoto, Japan). The cDNA was constructed using a High Capacity RNA-to-cDNA kit (Applied Biosystems, Foster City, CA, USA). Quantitative RT-PCR was performed using the TaqMan method in the Step-One-Plus RT-PCR System (Applied Biosystems). The primer sets for the target genes were TNF-α (Mm00443258_m1), IL-6 (Mm00446190_m1), CD14 (Mm00438094_g1), TLR4 (Mm00445273_m1), MCP-1 (Mm00441242_m1), CPT-1 (Mm01231183_m1), SREBP-1c (Mm00550338_m1) and β-actin (Mm00607939_s1). Amplifications were performed starting with a 10-min template denaturation step at 95 °C, followed by 40 cycles at 95 °C for 15 s and 60 °C for 1 min. The relative amounts of all of the RNA were normalized to the amount of β-actin, and the relative amounts of the RNAs were calculated using the comparative Cт method.

### 4.8. Statistical Analysis

All of the results are presented as the mean ± standard error. Statistical analyses were performed using the Statistical Analysis Systems package, Version 9.3 (SAS Institute, Cary, NY, USA). One-way analysis of variance (ANOVA) was used to compare three independent control groups (NC, AC and AH groups) with the *post hoc* Dunnet test. Two-way analysis of variance (ANOVA) was used to compare independent groups (AC, ACL, ACH, AH, AHL and AHH groups) with the *post hoc* Duncan multiple comparison test. *p* < 0.05 was accepted as statistically significance.

## 5. Conclusions

The overall data suggests that AK, at least at a high dose, may have the potential to attenuate ALD combined with an HFD by accelerating lipid β-oxidation and alcohol metabolism in the liver and also by suppressing inflammatory responses in the gut-liver axis. This is important, since people frequently drink alcohol with high-calorie foods in modern societies, and this combination may magnify the problem. The limitation of this study is that we could not identify the causative compound(s) responsible for the protective effect against hepatic steatosis. Future work should include studies on the chemical fingerprinting of AK. Studies should also include the evaluation of whether an array of components in AK can have a synergistic or an antagonistic effect on liver health. Even with this limitation, the results obtained in this study can be used as scientific background to support the use of AK as a functional food or as a food supplement to protect the liver against excessive intake of alcohol and high-fat foods.

## Figures and Tables

**Table 1:** Effects of AK on food intake and body weight of C57BL/6J mice fed with alcohol and HFD.

Groups	NC	Alcohol Ingestion Group					
		AC	ACL	ACH	AH	AHL	AHH
Food intake	2.73 ± 0.04	2.49 ± 0.12 ^a^	2.34 ± 0.09 ^a^	2.34 ± 0.05 ^a^	2.05 ± 0.04 ^b,^***	1.93 ± 0.02 ^b^	1.86 ± 0.04 ^b^
Initial body weight	22.83 ± 0.48	22.73 ± 0.51	23.15 ± 0.43	22.91 ± 0.60	23.14 ± 0.41	23.16 ± 0.34	23.22 ± 0.37
Final body weight	24.43 ± 0.26	24.07 ± 0.40	23.49 ± 0.38	22.64 ± 0.31	24.01 ± 0.24	23.50 ± 0.31	23.61 ± 0.28

Values expressed as the mean ± S.E. NC, normal control; AC, alcohol control; ACL, low-dose (1%) AK in alcohol group; ACH, high dose (3%) AK in alcohol group; AHC, alcohol/HFD control; AHL, low-dose AK in alcohol/HFD group; and AHH, high-dose AK in alcohol/HFD group. AK, ethanolic extract of *Acanthopanax koreanum* Nakai; HFD, high-fat diet. Values with different alphabets in each raw are significantly different among alcohol ingestion groups at *p* < 0.05 with Duncan’s multiple comparison tests. *** *p* < 0.0001 (AH to NC).

## Abbreviations

The following abbreviations are used in this manuscript:

ADH: alcohol dehydrogenase
AK: *Acanthopanax koreanum* Nakai
ALD: alcoholic liver disease
ALDH: acetaldehyde dehydrogenase
ALT: alanine aminotransferase
AST: aspartate aminotransferase
CAT: catalase
CD14: cluster of differentiation 14
CPT-1α: carnitine palmitoyltransferase 1α
CYP2E1: cytochrome P450 2E1
FAS: fatty acid synthase
HFD: high-fat diet
MCP-1: monocyte chemotactic protein-1
MyD88: myeloid differentiation factor 88
NAFLD: nonalcoholic fatty liver disease
NF-κB: nuclear factor kappa-light-chain-enhancer of activated B cells
IL-6: interluekin-6
LPS: lipopolysaccharide
ROS: reactive oxygen species
SREBP-1c: sterol regulatory element-binding protein-1c
STAT-3: signal transducer and activator of transcription-3
TC: total cholesterol
TG: triglyceride
TLR4: toll-like receptor 4
TNF-α: tumor necrosis factor- α
TRIF: TIR domain-containing adapter-inducing interferon

## References

[1] Ramaiah S., Rivera C., Arteel G. (2004). Early-phase alcoholic liver disease: An update on animal models, pathology, and pathogenesis. Int. J. Toxicol..

[2] Rutkowski J.M., Stern J.H., Scherer P.E. (2015). The cell biology of fat expansion. J. Cell Bio..

[3] Beier J.I., Arteel G.E., McClain C.J. (2011). Advances in alcoholic liver disease. Curr Gastroenterol. Rep..

[4] Demori I., Voci A., Fugassa E., Burlando B. (2006). Combined effects of high-fat diet and ethanol induce oxidative stress in rat liver. Alcohol.

[5] Lee I.S., Park S., Park K., Choue R. (2011). Hepatoprotective activity of scutellariae radix extract in mice fed a high fat diet with chronic alcohol exposure. Phytother. Res..

[6] Gäbele E., Dostert K., Dorn C., Patsenker E., Stickel F., Hellerbrand C. (2011). A new model of interactive effects of alcohol and high-fat diet on hepatic fibrosis. Alcohol Clin. Exp. Res..

[7] Nan J.X., Park E.J., Nam J.B., Zhao Y.Z., Cai X.F., Kim Y.H., Sohn D.H., Lee J.J. (2004). Effect of Acanthopanax koreanum Nakai (Araliaceae) on d-galactosamine and lipopolysaccharide-induced fulminant hepatitis. J. Ethnopharmacol..

[8] Nan J.X., Jin X.J., Lian L.H., Cai X.F., Jiang Y.Z., Jin H.R., Lee J.J. (2008). A diterpenoid acanthoic acid from Acanthopanax koreanum protects against d-galactosamine/lipopolysaccharide-induced fulminant hepatic failure in mice. Biol. Pharm. Bull..

[9] Kang J.S., Linh P.T., Cai X.F., Kim H.S., Lee J.J., Kim Y.H. (2001). Quantitative determination of eleutheroside B and E from Acanthopanax species by high performance liquid chromatography. Arch. Pharm. Res..

[10] Phuong N.T., Lee K.A., Jeong S.J., Fu C.X., Choi J.K., Kim Y.H., Kang J.S. (2006). Capillary electrophoretic method for the determination of diterpenoid isomers in Acanthopanax species. J. Pharm. Biomed. Anal..

[11] Yamazaki T., Shimosaka S., Sasaki H., Matsumura T., Tukiyama T., Tokiwa T. (2007). (+)-Syringaresinol-di-*O*-beta-d-glucoside, a phenolic compound from Acanthopanax senticosus Harms, suppresses proinflammatory mediators in SW982 human synovial sarcoma cells by inhibiting activating protein-1 and/or nuclear factor-kappaB activities. Toxicol. In Vitro.

[12] Nhiem N.X., Tung N.H., Kiem P.V., Minh C.V., Ding Y., Hyun J.H., Kang H.K., Kim Y.H. (2009). Lupane triterpene glycosides from leave of Acanthopanax koreanum and their cytotoxic activity. Chem. Pharm. Bull. (Tokyo).

[13] Schmolz M.W., Sacher F., Aicher B. (2001). The synthesis of Rantes, G-CSF, IL-4, IL-5, IL-6, IL-12 and IL-13 in human whole-blood cultures is modulated by an extract from *Eleutherococcus senticosus* L. roots. Phytother. Res..

[14] Kim K.J., Hong H.D., Lee O.H., Lee B.Y. (2010). The effects of Acanthopanax senticosus on global hepatic gene expression in rats subjected to heat environmental stress. Toxicology.

[15] Wu Y.L., Jiang Y.Z., Jin X.J., Lian L.H., Piao J.Y., Wan Y., Jin H.R., Joon Lee J., Nan J.X. (2010). Acanthoic acid, a diterpene in Acanthopanax koreanum, protects acetaminophen-induced hepatic toxicity in mice. Phytomedicine.

[16] Yang Y.K., Lin W., Kwon O. (2014). Protective effects of Acanthopanax koreanum Kakai extract against carbon tetrachloride-induced liver injury in Sprague-Dawley rats. J. Nutr. Health.

[17] Jung M.G., Do G.M., Shin J.H., Ham Y.M., Park S.Y., Kwon O. (2013). *Acanthopanax koreanum* Nakai modulates the immune response by inhibiting TLR 4-dependent cytokine production in rat model of endotoxic shock. Nutr. Res. Pract..

[18] Su-Hong C., Qi C., Bo L., Jian-Li G., Jie S., Gui-Yuan L. (2015). Antihypertensive Effect of Radix Paeoniae Alba in Spontaneously Hypertensive Rats and Excessive Alcohol Intake and High Fat Diet Induced Hypertensive Rats. Evid. Based. Complement. Altern Med..

[19] Tahir M., Rehman M.U., Lateef A., Khan R., Khan A.Q., Qamar W., Ali F., O'Hamiza O., Sultana S. (2013). Diosmin protects against ethanol-induced hepatic injury via alleviation of inflammation and regulation of TNF-alpha and NF-kappaB activation. Alcohol.

[20] Botros M., Sikaris K.A. (2013). The de ritis ratio: The test of time. Clin. Biochem. Rev..

[21] Purohit V., Gao B., Song B.J. (2009). Molecular mechanisms of alcoholic fatty liver. Alcohol. Clin. Exp. Res..

[22] Nebert D.W., Dalton T.P. (2006). The role of cytochrome P450 enzymes in endogenous signalling pathways and environmental carcinogenesis. Nat. Rev. Cancer.

[23] Edenberg H.J. (2007). The genetics of alcohol metabolism: Role of alcohol dehydrogenase and aldehyde dehydrogenase variants. Alcohol Res Health.

[24] Hung H.C., Chuang J., Chien Y.C., Chern H.D., Chiang C.P., Kuo Y.S., Hildesheim A., Chen C.J. (1997). Genetic polymorphisms of CYP2E1, GSTM1, and GSTT1; environmental factors and risk of oral cancer. Cancer Epidemiol. Biomark. Prev..

[25] Maher J. (2001). The CYP2E1 knockout delivers another punch: First ASH, now NASH. Alcoholic steatohepatitis. Nonalcoholic steatohepatitis. Hepatology.

[26] Leung T.M., Nieto N. (2013). CYP2E1 and oxidant stress in alcoholic and non-alcoholic fatty liver disease. J. Hepatol..

[27] Handler J.A., Thurman R.G. (1990). Redox interactions between catalase and alcohol dehydrogenase pathways of ethanol metabolism in the perfused rat liver. J. Biol. Chem..

[28] Szabo G., Bala S. (2010). Alcoholic liver disease and the gut-liver axis. World J. Gastroenterol..

[29] Gäbele E., Brenner D.A., Rippe R.A. (2003). Liver fibrosis: Signals leading to the amplification of the fibrogenic hepatic stellate cell. Front Biosci..

[30] Kleiner D.E., Brunt E.M., van Natta M., Behling C., Contos M.J., Cummings O.W., Ferrell L.D., Liu Y.C., Torbenson M.S., Unalp-Arida A. (2005). Design and validation of a histological scoring system for nonalcoholic fatty liver disease. Hepatology.

[31] Volkl A., Fahimi H.D. (1998). Isolation of peroxisomes. Cell Biol. A Lab. Handb..

[32] Bonnichsen R.K., Brink N.G. (1955). [78] Liver alcohol dehydrogenase. Methods Enzymol..

[33] Koivula T., Koivusalo M. (1975). Different forms of rat liver aldehyde dehydrogenase and their subcellular distribution. Biochim. Biophys. Acta.

[34] Johansson L.H., Borg L.H. (1988). A spectrophotometric method for determination of catalase activity in small tissue samples. Anal. Biochem..

